# Differential expression of two novel odorant receptors in the locust (*Locusta migratoria*)

**DOI:** 10.1186/1471-2202-14-50

**Published:** 2013-04-22

**Authors:** Haozhi Xu, Mei Guo, Ying Yang, Yinwei You, Long Zhang

**Affiliations:** 1 Department of Entomology, China Agricultural University, Beijing 100193, P. R. China; 2 Current Address: Research Program of Center for DNA Typing, Department of Biochemistry and Molecular Biology, Fourth Military Medical University, Xi’an 710032, P. R. China; 3 High-tech Research Center, Shandong Academy of Agricultural Sciences, Jinan 250100, China

**Keywords:** Odorant receptor, Locust, Odorant receptor neuron, Basiconic sensillum, Expression

## Abstract

**Background:**

Olfaction in animals is important for host localization, mating and reproduction in heterogeneous chemical environments. Studying the molecular basis of olfactory receptor neurons (ORNs) systems can elucidate the evolution of olfaction and associated behaviours. Odorant receptors (ORs) in insects have been identified, particularly in the holometabolous model *Drosophila*, and some of them have been functionally studied. However, ORs in the locust—a hemimetabolous model insect and the most important insect crop pest—have not yet been identified, hindering our understanding of locust olfaction. Here, we report for the first time four putative ORs in *Locusta migratoria*: *Lmig*OR1, *Lmig*OR2, *Lmig*OR3 and *Lmig*OR4.

**Results:**

These four putative OR genes encoded proteins with amino acids of 478, 436, 413 and 403 respectively. Sequence identity among them ranged from 19.7% to 35.4%. All ORs were tissue-specifically expressed in olfactory organs, without sex-biased characteristics. However, *LmigOR*1, *LmigOR*3 and *LmigOR*4 were only expressed in the antenna, while *LmigOR*2 could also be detected in mouthparts. *In situ* hybridization demonstrated that the *LmigOR*1antisense probe labelled olfactory receptor neurons (ORNs) in almost all segments of the antenna, but only a few segments housed ORNs expressing *LmigOR*2. The number of neurons labelled by *LmigOR*1 antisense probes in each antennal segment was much greater (>10 neurons/segment) than that labelled by *LmigOR*2 probes (generally 1–3 neurons/segment). Furthermore, some of the labelled neurons could be attributed to the basiconic sensilla, but *LmigOR*1 and *LmigOR*2 were expressed in different subtypes.

**Conclusions:**

Our results strongly suggested that these newly discovered genes encode locust ORs and the differential expression patterns of *LmigOR*1 and *LmigOR*2 implied distinct functions. These results may offer insights into locust olfaction and contribute to the understanding of the evolution of insect chemoreception.

## Background

Mammals and insects have adapted evolutionarily to the heterogeneous chemical environments in which they live. Odorant receptors (ORs) in ORNs systems are involved in scent detection and discrimination and are therefore key to understanding the molecular evolution of olfactory mechanisms in animals [1-4].

Insect ORs are evolutionarily unrelated to their vertebrate counterparts. Although insect ORs possess seven transmembrane domains like the G-protein-coupled ORs in vertebrates, the transmembrane topology is completely inverted [5-7]. Since the discovery of the first OR in *Drosophila* through bioinformatics analysis of the partially sequenced genome [2,8,9], numerous OR-coding genes have been identified in various holometabolous insects. In the genomes characterized to date, 60 OR genes have been found in Drosophila [10,11], 79 in mosquito [11], 162 in honey bee [12], and 341 in red floor beetle [13]. Insect ORs evolve rapidly, and there is considerable sequence diversity among OR proteins—many show only ~20% similarity [14]. The olfactory systems of a variety of insect species have been extensively studied. However, it is still very difficult to draw satisfying conclusions about the evolution of insect olfaction because of the absence of studies on some important taxa, such as orthopteran insects [15].

Most insect ORs are only expressed in olfactory organs such as antennae or maxillary palps [16-19]. Aside from the highly conserved odorant receptor co-receptor (ORco) subfamily, the expression of each individual OR is confined to a unique subset of ORNs, resulting in molecular diversity among ORNs. The “One-Receptor-One-Neuron” model proposed for mammalian olfactory systems also applies to most insect ORNs [2,20]. ORNs expressing the same ORs were housed in electrophysiologically identical sensilla subtypes and converged to the same glomerulus in the antennal lobe. Extracellular single-unit recordings from individual olfactory sensilla have revealed that different odorants elicit responses from different subsets of ORNs, and that ORNs exhibit a remarkable diversity of response properties [3,4,21]. ORNs housed in different sensilla types expressed distinct ORs, allowing the sensilla to be characterized by their molecular and cellular properties [2,4,19,21-23].

Locust (*Locusta migratoria*) is regarded as a model animal of hemimetabolous insects, and is a notorious worldwide pest that has historically caused tremendous damage to agricultural production [24]. Its behaviours, such as feeding, migration, mating, defence, aggregation, and reproduction, are probably mediated by chemoreception. The development of alternative control methods to replace chemical pesticides will depend on understanding the molecular mechanisms that regulate locust behaviour.

Thus far, we have identified and characterized several locust odorant-binding proteins (OBPs) and chemosensory proteins (CSPs) that are thought to be involved in chemoreception in insects [25-29]. Recently, the evolutionally conserved ORco was identified in locust and found to be ubiquitously expressed in ORNs, just as in other insects [30]. However, extensive efforts to identify functionally specific ORs have failed because of the low sequence homology between ORs and the large evolutionary distances among insect clades. The absence of knowledge about ORs that transduce the binding of odorants into neural activity has hindered further understanding of locust olfaction.

Here, we have identified four novel OR-coding genes in locust and found that *LmigOR*1 and *LmigOR*2 showed differential expression patterns in olfactory organs. *LmigOR*1 was specifically expressed in antennae, whereas *LmigOR*2 transcripts could also be detected in mouthparts. Some of the ORNs expressing *LmigOR*1 or *LmigOR*2 could be found in the basiconic sensilla, but the receptors were present in different sensilla subtypes. These results may offer insights into locust olfaction and contribute to the understanding of the evolution of insect chemoreception.

## Results

### Identification of odorant receptors in locust

**Figure 1:**
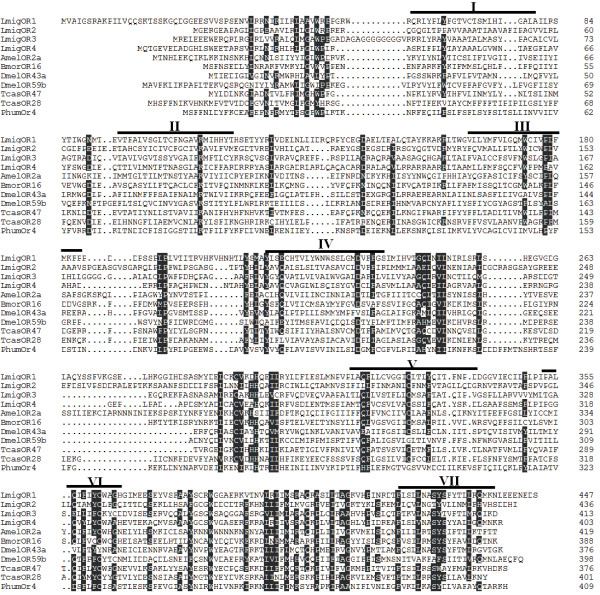
**Amino acid sequences alignment of newly identified ORs in locust to odorant receptors from other insects.** Residues conserved in >50% of the predicted proteins are shaded. The numbers to the right refer to the position of the last residue of each line. The positions of putative transmembrane domains (I–VII), which were predicted based on *Lmig*OR1, are indicated with black bars. Accession numbers for the selected odorant receptors: *Tcas*OR28: [GeneBank: EEZ99241]; *Tcas*OR47: [GeneBank: EFA02940]; *Amel*OR2a: [GeneBank: XP_003250826]; *Bmor*OR16: [GeneBank: NP_001104832]; *Phum*OR4: [GeneBank: XP_002427433]; *Dmel*OR43a: [GeneBank: ADK48470]; *Dmel*OR59a: [GeneBank: AAF47008].

**Figure 2:**
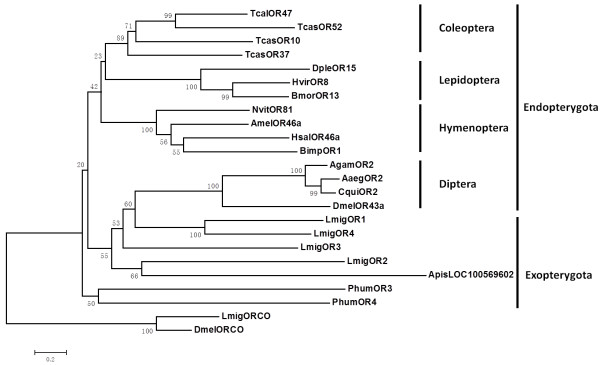
**Phylogenetic relationships of *Lmig*ORs with odorant receptors from other insects.** This distance tree was rooted by declaring the ORco as the outgroup. More closely related insects ORs were removed to facilitate phylogenetic analysis and representation; therefore, many of the lineage-specific subfamily expansions in these insects are not obvious. Accession number for the selected odorant receptors: *Hsal*46a: [Genebank: EFN79914]; *Apis*OR46a: [Genebank: XP_003249621]; *Bimp*OR1: [Genebank: XP_003487625]; *Nvit*OR81: [Genebank: NP_001164394]; *Dple*OR15: [Genebank: EHJ72224]; *Hvir*OR8: [Genebank: CAD31949]; *Bmor*OR13: [Genebank: NP_001166603]; *Tcas*OR37: [Genebank: EEZ99229]; *Tcas*OR10: [Genebank: EFA09294]; *Tcas*OR47: [Genebank: EFA02940]; *Tcas*OR52: [Genebank: EEZ99301]; *Dmel*OR43a: [Genebank: ADK48356]; *Agam*OR2: [Genebank: XP_310173]; *Aaeg*OR2: [Genebank: XP_001651755]; *Cqui*OR2: [Genebank: XP_001864544]; *Apis*LOC100569602: [Genebank: XP_003243258]; *Phum*OR3: [Genebank: XP_002429370]; *Phum*OR4: [Genebank: XP_002427433]; *Lmig*ORco: [Genebank: JN989549]; *Dmel*ORco: [Genebank: AAF52031].

A tBlastn search of the locust antenna expressed sequence tag (EST) database, using previously identified insect ORs as queries, identified 2 sequence fragments coding putative ORs. Full cDNA length was obtained using RACE. Iterative blast using the newly identified ORs as query sequences identified another two putative ORs fragments and their full length was obtained by walking sequencing of the clones. The complete coding sequences were deposited in GenBank and designated as *Lmig*OR1 [Genebank: JQ766965], *Lmig*OR2 [Genebank: JQ766966], *Lmig*OR3 [Genebank: KC689310] and *Lmig*OR4 [Genebank: KC689311]. They consisted of amino acids of 478, 436, 413 and 403. The theoretical molecular weights were 50.762, 47.867, 44.321 and 44.805 KD respectively. Sequence identities among them ranged from 19.7% to 35.4% (Table 1). A Pfam search clearly classified them into the seven-transmembrane (7-TM) superfamily, although TMHMM prediction gave relatively low probabilities for the seventh transmembrane domains, perhaps in part because of ambiguities in the hydrophobic regions. The predicted locations of putative transmembrane domains within the sequences are indicated in Figure 1. Phylogenetic analysis of several more closely related insect ORs confidently grouped *Lmig*ORs into a monophyletic lineage, indicating they can be as designated as locust-specific ORs (Figure 2).

**Table 1:** Amino acids identities among the four putative locust ORs

	***Lmig*OR1**	***Lmig*OR2**	***Lmig*OR3**	***Lmig*OR4**
***Lmig*OR1**	100%			
***Lmig*OR2**	19.6%	100%		
***Lmig*OR3**	21.7%	19.9%	100%	
***Lmig*OR4**	35.4%	20.7%	25.3%	100%

### The temporal and spatial expression profile of *LmigOR*s

**Figure 3:**
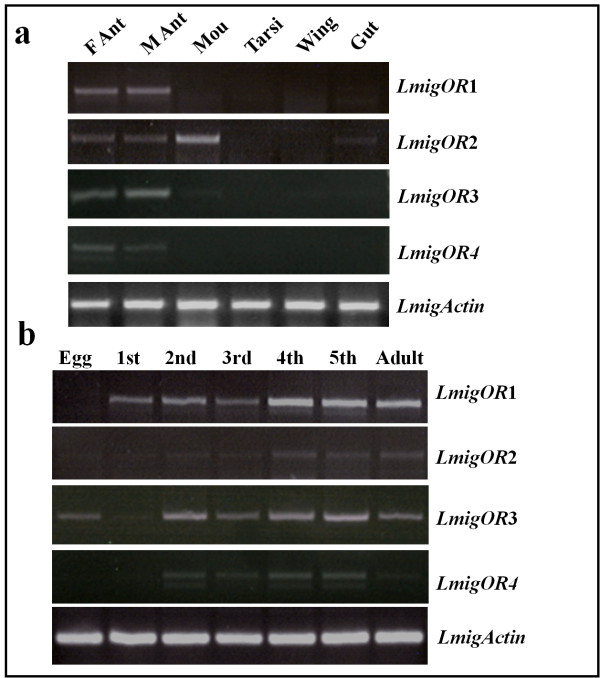
**Spatial and temporal expression patterns of *LmigOR*s. a**, Tissue-specific expression of *LmigOR*s in olfactory organs. M Ant, male antenna; F Ant, female antenna; Mou, mouthparts, Tarsi, locust tarsi; Wing, locust wings; Gut, locust gut. **b**, Dynamical expression of *LmigOR*s in antenna during locust development. Egg, locust eggs; 1st–5th, locust antenna of nymphs from 1st to 5th instar; Adult, locust adult antenna. The locust actin gene was used as control for the integrity of the cDNA template. Amplification products were analysed on agarose gels and visualized by UV illumination after ethidium bromide staining. All tissues were dissected from gregarious locusts unless indicated.

To assess the tissue-specific expression patterns of the ORs in *Locusta migratoria*, polymerase chain reaction (PCR) experiments were performed using sequence-specific primers that amplified ~700-bp sequences from cDNA pools produced from 1 μg of total RNA. The *LmigOR*1, *LmigOR*3 and *LmigOR4* mRNA were detected exclusively in locust antennae, whereas the *LmigOR*2 transcripts were also abundantly detected in mouthparts. In non-olfactory organs, such as tarsi, wings and guts, we did not detect any specific expression, although numerous gustation-related chemosensilla (chaetic sensilla) were present. We found no differences in tissue distribution between sexes (Figure 3a). Interestingly, we found that the expression levels of *LmigOR*1 and *LmigOR*2 in the antennae increased with age, especially after the fourth instar stage. In contrast, *LmigOR*4 expressed more highly in nymph than in adult. Among these genes, only *LmigOR*3 could be detected in eggs besides its abundant expression from nymph to adult (Figure 3b). The locust actin gene was constitutively expressed at high levels in all tissues at all developmental stages, providing a control for the integrity of the cDNA template (Figure 3).

### Expression of *LmigOR*1 and *LmigOR*2 in ORNs in olfactory organs

**Figure 4:**
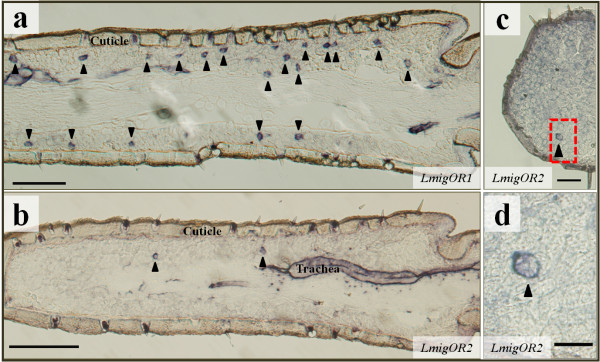
**Cellular localization of *LmigOR*1 and *LmigOR*2 in olfactory organs. a**, Overview of *LmigOR1*-expressing cells in a locust antennal segment. **b**, Overview of *LmigOR*2-expressing cells in a locust antennal segment. **c**, Cellular localization of *LmigOR*2 transcripts in maxillary palps. **d**, A close view of the boxed area in **c** showing palpal cells expressing *LmigOR*2. Arrowheads indicate cells expressing *LmigOR*1 (**a**) and *LmigOR*2 (**b**–**d**). Scale bars: **a**, **b**: 100 μm; **c**: 50 μm; **d**: 20 μm.

To determine whether the *LmigORs* genes were specifically expressed in ORNs, we carried out *in situ* hybridization using gene-specific probes. We found that only a small subset of the antennal cells present in each section of adult antenna was labelled by the *LmigOR*1 antisense probe. We found that more than 10 labelled cells could be detected in each segment (Figure 4a). By contrast, cells labelled by the *LmigOR*2 antisense probe were found in both antennae and maxillary palps. The number of cells/segment was about 1–3 cells—much less than that for *LmigOR*1 (Figure 4b–d). We did not detect any labelled antennal cells expressing *LmigOR*3 or *LmigOR*4 (data not shown).

**Figure 5:**
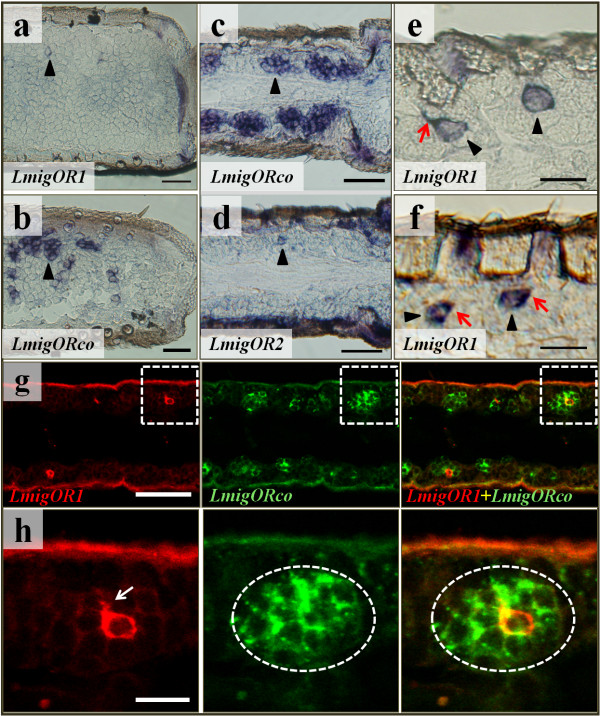
**Neuronal identity of antennal cells expressing *LmigOR*s. a**–**b**, The labelling pattern of *LmigOR*1 (**a**) and *LmigORco* (**b**) antisense probe on consecutive sections of locust antenna. **c**–**d**, The labelling pattern of *Lmig*ORco (**c**) and *LmigOR*2 (**d**) antisense probe on consecutive sections of locust antenna. **e**–**f**, Illustration of occasionally labelled dendritic like structures (indicated by red arrows). **g**, Two-colour *in situ* hybridization was performed on longitudinal antennal sections to illustrate the expression of *LmigOR*1 (Red) and *LmigORco* (Green). Localization of *LmigOR*1 expressing cells in cell clusters expressing *LmigORco* confirmed its neural identity. **h**, Close view of boxed areas in **g**. Occasionally labelled dendritic like structures were indicated by arrow. Circled areas indicate ORNs cluster expressing *LmigORco* and sharing the same sensillum. Scale bar: **a**–**d**, **g**: 50 μm; **e**, **f**, **h**: 20 μm.

To confirm the neuronal identity of the labelled cells, we performed RNA *in situ* hybridization on consecutive sections using RNA probes for *LmigOR*1/2 and *LmigORco.* The results showed that antennal cells expressing *LmigOR*1/2 located within ORNs clusters expressing *LmigORco* (Figure 5a-d), indicating the putative *LmigOR*1 and *LmigOR*2 expressed in ORNs. This was further verified by two-color *in situ* hybridization using fluorophore labelled probes (Figure 5g, h). We sometimes observed labelling of, not only the cell body, but also the dendritic like structure (Figure 5e, f, h), further identifying these labelled cells as ORNs.

### *LmigOR*1 and *LmigOR*2 map to distinct subtypes of the basiconic sensilla

**Figure 6:**
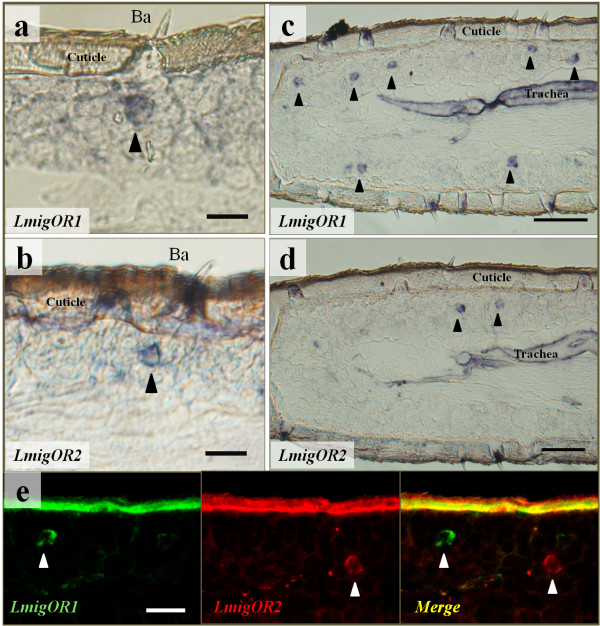
***LmigOR*1 and *LmigOR*2 expressed in ORNs housed by basiconic sensilla. a**–**b**, Basiconic sensillum housed ORNs expressing *LmigOR*1 (**a**) and *LmigOR*2 (**b**). **c**–**e**, The expression of *LmigOR*1 and *LmigOR*2 in distinct subset of antennal ORNs was verified on consecutive sections (**c**–**d**) and by two-color *in situ* hybridization (**e**). Fluorescent signals were visualized using detection systems indicating *LmigOR*1-labelled neurons by green fluorescence and *LmigOR*2 positive cells by red fuorescence (**e**). Arrowheads denote antennal cells expressing *LmigOR*1 (**a**, **c**, **e**) and *LmigOR*2 (**b**, **d**, **e**). Ba: basiconic sensillum. Scale bar: **a**, **b**, **e**: 20 μm; **c**, **d**: 50 μm.

We then carried out an imaging experiment to assign the labelled ORNs to morphologically specific sensillum types. The results demonstrated that some ORNs expressing *LmigOR*1 and *LmigOR*2 could be unambiguously attributed to basiconic sensilla (Figure 6a, b). In contrast, we did not observe any neurons labelled with *LmigOR*1 or *LmigOR*2 probes in trichoid, coeloconic, or chaetic sensilla. No positive signals could be detected when the sense probe was applied (data not shown). Unlike the colocalization pattern seen with *LmigORco, LmigOR1* and *LmigOR2* were expressed in discrete subset of ORNs (Figure 6c–e), indicating they were present in different sensilla subtypes.

## Discussion

We have identified four putative ORs in *Locusta migratoria*, named as *Lmig*OR1, *Lmig*OR2, *Lmig*OR3 and *Lmig*OR4 and found that they harboured all the characteristic features of insect ORs: seven trans-membrane domains and conserved Ser-Tyr-Ser motif, expression in olfactory organs, and more conserved amino acids at the C terminus than in the N terminal region [2,8,17,31]. However, the new ORs were highly divergent from known insect ORs. This is consistent with the requirement for recognition of a large number of molecularly diverse odorants, and with species-specific expansion of insect OR gene subfamily lineages. Locust has the special microglomerular systems innervated by branched ORNs and projection neurons and may not follow the law of one receptor to one glomerulus in insect [32]. The complex organization of the olfactory system in locust might increase coding capacity and this was correlated with the complicated behavioural plasticity of the locust. Our analysis of the phylogenetic relationships of locust ORs with ORs from other insects further agreed with the view that orthopteran insects, including the locust, play a special role in the study of olfactory evolution, particularly in invertebrates [15].

ORco is generally expressed in insect ORNs and may serve as a marker for ORNs [3,30,33]. Our observation that a few antennal cells expressing *LmigOR*1 were located within clusters of *LmigORco-*expressing cells unambiguously defined them as ORNs. The specific expression of *LmigOR*1 and *LmigOR*2 in olfactory organs indicated that they are involved in olfaction. Although both OR transcripts could be detected in antennae, their labelling patterns were different. ORNs expressing individual genes were restricted to distinct subsets of antennal ORNs. The assignment of the two OR genes to different functional types of basiconic sensilla indicate that they have contrasting response profiles. The failure of our attempts to localize the expression of *LmigOR*3 and *LmigOR*4 in antenna using RNA *in situ* hybridization experiments may be partially caused by the high GC content of these *OR*s, which was 71.6% and 67% respectively.

Insects are also equipped with a second nose: the palps. These organs in hawk moth and mosquito serve as detectors for CO_2_[34,35], while in *Drosophila*, they may play a role in taste enhancement [36]. Locust palps have been shown to play critical roles for locating and evaluating food resources [37]. It was therefore not surprising that the expression of *LmigOR*2 was detected in mouthparts. However, the most fascinating finding was that *LmigOR*2 was expressed in both antenna and palps. ORs in *Drosophila* are selectively expressed in only one of these organs [16,38,39]. In *Drosophila*, ORNs housed by basiconic sensilla on antennae and maxillary palpi expressed discrete subsets of OR genes and projected to distinct regions of the antennal lobe, indicating different functions [39,40]. In locust, the external morphology of the basiconic sensilla on maxillary palpi resembles that on antennae, except for a prominent socket in connection with a membranous cuticle [28]. Whether the basiconic sensilla on maxillary palpi and antennae have different physiological functions must be experimentally verified. Nevertheless, the overlapping expression of *LmigOR*2 on antennae and palpi, which resembles the differential expression of some mosquito ORs across all three olfactory appendages, reflected a topographic ordering of sensitivity [34,41].

Some odorant binding proteins, such as LUSH, may serve as triggers that activate ORs through conformational change upon odorant binding [42]. In locust, the odorant-binding protein *Lmig*OBP1 was expressed in all basiconic sensilla on antennae and palps [28,43]. Its coexpression with *LmigOR*s in the same sensilla made it reasonable to guess that these two proteins may interact; this awaits further investigation.

We can only tentatively deduce the functions of *LmigOR*s because of their high sequence divergence from known ORs. Nevertheless, the differential expression of *LmigOR*1 and *LmigOR*2 indicates distinct functions. Furthermore, their ubiquitous expression during development and sex-independent expression pattern suggests that these two receptors may be involved in the detection of general odours rather than pheromones. The *LmigOR*2 is more likely to be involved in feeding because of its abundant expression in mouthparts [43,44].

## Conclusions

We have for the first time identified four novel OR-coding genes in locust, named as *LmigOR1*, *LmigOR2, LmigOR3* and *LmigOR4*. Their encoded proteins consist of amino acids of 478, 436, 413 and 403; and their theoretical molecular weights were 50.762, 47.867, 44.321 and 44.805 KD respectively. Sequence identities among them ranged from 19.7% to 35.4%. Our analysis of the phylogenetic relationships of locust ORs with ORs from other insects further agreed with the view that orthopteran insects, including the locust, play a special role in the study of olfactory evolution, particularly in invertebrates. They all specifically express in olfaction related organs, antenna or mouthparts in locust. *LmigOR*1 and *LmigOR*2 showed differential expression patterns in olfactory organs. *LmigOR*1 was specifically expressed in antennae, whereas *LmigOR*2 transcripts could also be detected in mouthparts. Some of the ORNs expressing *LmigOR*1 or *LmigOR*2 could be found in the basiconic sensilla, but the receptors were present in different sensilla subtypes. These results may offer insights into locust olfaction and contribute to the understanding of the evolution of insect chemoreception.

## Methods

### Insects

Locusts (*L. migratoria*) were obtained from the Department of Entomology, China Agricultural University, Beijing, and raised in crowded conditions at 28–30°C, with 60% relative humidity, and a light:dark photoperiod of 18:6 h. They were fed daily with fresh wheat shoots. Intact antennae, mouthparts, tarsi, wings, and midguts were dissected using forceps and stored at −80°C until further processing.

### cDNA Library construction

An antennal cDNA library of fourth-instar nymphal locusts was constructed using the SuperScript® Full Length cDNA Library Construction Kit II (Invitrogen, Grand Island, NY, USA) following the manufacturer’s protocol. Highly abundant transcripts were subtracted using the genome-saturation hybridization procedure [45]. Sequencing of ~10^4^ randomly selected positive clones was performed using an ABI 3730XL capillary sequencer (Invitrogen).

### Identification of putative *LmigOR*s-coding genes and sequence analysis

Vector sequences were detected and masked using Cross_Match. Assembly of clean ESTs into contigs was performed using the Phrap software package (http://www.phrap.org/phredphrapconsed.html). Previously identified insect OR-coding genes were downloaded from NCBI and used as queries to identify putative locust ORs in the formatted EST database by tBlastn searches with the blast-2.2.25+ package (http://ftp.ncbi.nlm.nih.gov/blast/executables/blast+/2.2.25/). Newly identified ORs were used as query sequences across the database to identify others iteratively. For transmembrane domain predictions, the TMHMM program (v. 2.0) (http://www.cbs.dtu.dk/services/TMHMM-2.0/) was used. Protein sequence alignment was performed in DNAMAN version 7. An unrooted consensus neighbour-joining tree was calculated using default settings with pairwise gap deletions in MEGA-5 [46]. Branch support was assessed using 1,000 bootstrap replicates.

### Rapid amplification of cDNA ends (RACE)

The gene fragments were extended in both 5′ and 3′ directions for *LmigOR*1 and 3′ directions for *LmigOR*2 by RACE-PCR with gene-specific primers in conjunction with a SMART-amplified antennal cDNA and SMART adapter-specific primers using the Smarter RACE Kit (Clontech, Palo Alto, CA, USA) according to the manufacturer’s manual. Based on the partial *LmigOR*s sequences obtained by blast search of the cDNA library, specific primers for RACE-PCR were designed for touchdown PCRs. PCR products were gel-purified and subcloned using the pGEM-T Easy Kit for sequencing (Promega, Madison, WI, USA).

### Expression of *LmigOR*s in different tissues and developmental stages

Total RNA was isolated from frozen tissues using Trizol reagent (Invitrogen) following the manufacturer’s protocols. Reverse transcription was performed using the Quant cDNA Synthesis Kit (Tiangen, Beijing, China) with 1 μg unpurified total RNA as template. Non-quantitative RT-PCRs were performed with gene-specific primers. To test the integrity of the cDNA preparation, primers for the *L. migratoria* actin gene [Genebank: AY344445] were used. PCR products were run on 1.2% agarose gels and visualized by ethidium bromide staining.

### Probe preparation and *in situ* hybridization

Templates of both ORs were generated by standard PCR using gene-specific primer pairs. Digoxigenin (DIG)- or Biotin- labelled antisense and sense probes were generated from linearized recombinant pGem-T Easy plasmids using the T7/SP6 RNA transcription system (Roche, Basel, Switzerland) following recommended protocols. RNA probes were subsequently fragmented to an average length of about 300 bp by incubation in carbonate buffer. RNA *in situ* hybridization was performed according to previously reported procedures [30]. Briefly, antennae were dissected, embedded in the freezing medium (Tissue-Tek O.C.T. Compound; Sakura Finetek Europe, Zoeterwoude, Netherlands). Sections (12 μm) were prepared at −24°C using a Jung CM300 cryostat (Leica, Nussloch, Germany) and thaw-mounted on SuperFrost Plus slides (Boster, Wuhan, China). After series of fixing and washing procedures, 100 μl hybridization solution (Boster) containing RNA probe was placed onto the tissue section. After adding a coverslip, slides were incubated in a humid box at 55°C overnight. After hybridization, slides were washed twice for 30 min in 0.1 × saline-sodium citrate (SSC) at 60°C, treated with 1% blocking reagent (Roche) in TBST for 30 min at room temperature, then incubated for 30 min with an anti-digoxigenin alkaline phosphatase conjugated antibody (Roche,). Hybridization signals were visualized using NBT/BCIP substrate. Tissue sections were analysed on Olympus IX71microscope (Olympus, Tokyo, Japan).

Fluorescent RNA *in situ* hybridization was carried out in the same way using DIG- and/or biotin-labelled probes. DIG-labelled probes were visualized by the anti-DIG alkaline phosphatase-conjugated antibody in combination with HNPP/Fast Red (Roche). For biotin-labelled probes, TSA kit (Perkin Elmer, MA, USA), including a strepavidin horse radish peroxidase-conjugate and fluorescin-tyramides as substrate, were used. Images were captured on Olympus BX45 confocal microscope and analyzed using FV1000 software. Images were not altered except for adjusting the brightness or contrast uniformly within a single figure (Additional file 1: Table S2).

## Competing interests

The authors declare that they have no competing interest.

## Authors’ contribution

All authors designed the experiment together. HZX, MG, YY and YWY identified and characterized the expression of odorant receptors. HZX, MG and YY performed cellular localization studies, interpreted the results and produced the figures. LZ wrote the paper. All authors read and approved of the final manuscript.

## Supplementary Material

Additional file 1: Table S2 Primer sequences used in the present work. Click here for file
