# Supplementary material for: Differential expression of two novel odorant receptors in the locust (Locusta migratoria)
Source: BMC Neurosci. 2013 Apr 22;14:50. doi: 10.1186/1471-2202-14-50 (PMC3658887; doi:10.1186/1471-2202-14-50)
Supplement: Additional file 1: Table S2 — Primer sequences used in the present work. [file 1471-2202-14-50-S1.pdf]

Additional file 1: Table S2

Table 2 Primer sequences used in the present work

Name	Sequence
OR1-5RACE	CGCGTGATGGTGACGAGCGG
OR1-3RACE	CAACAGGGACACCTTCCTCTCGC
OR2-3RACE	GCCACGAGGGCCGAGTACGGCTCAG
OR1-s	TACACCCACAGCGAGACCTA
OR1-as	CATATTCCGCCCACGCAAAG
OR2-s	CACCTACCACCTGCTGTACG
OR2-as	CTACTGTGATCTCCACCGGC
OR3-s	TGCTTCTCCGTGTTCAACTG
OR3-as	AGCACCTTGGTGAAGGTCTG
OR4-s	TGCTTCTCCGTGTTCAACTG
OR4-as	TGCACAAACCTGCAAACTTC
Actin-s	GCAAAGCTGGCTTCGCCG
Actin-as	ATGTTCCTCGGGCGCCAC
OR1-probe-s	AAGGGGTGGGAGACGGCCTG
OR1-probe-as	CAGCTCCTCCCCAACGACAGC
OR2-probe-s	ATGGGTGAGCGTGGAGAGGC
OR2-probe-as	GGTCATCGCTGTGGACGTGG
OR3-probe-s	TCATGTTCTCGCAGTGCTTC
OR3-probe-as	TAGGATGCATTCACCAACGA
OR4-probe-s	GACGCTCCAGACAGCATGTA

OR4-probe-as	TAGGATGCATTCACCAACGA
